# Draft genome assemblies from seven Bacillaceae isolates from woodland soil

**DOI:** 10.1128/mra.00289-25

**Published:** 2025-05-20

**Authors:** Anna L. McLoon, Julia M. Barker, Gillian M. Churan, Anthony Cucca, Lillian Gardner, Justin Gejo, Francesca Gerbasi, Michaela Higgins, Lillian Kronau, Jalin Le, Alyssa Lunman, Kelly Maune, Veezen Denise Mondelo, Madeline Naef, Caitlin Rigby, Caitlin Spiliotis

**Affiliations:** 1 Department of Biology, Siena College 5173, Loudonville, New York, USA; DOE Joint Genome Institute, Berkeley, California, USA

**Keywords:** *Bacillus*, genomics, soil microbiology

## Abstract

We isolated seven endospore-forming bacteria from campus woodland and sequenced their genomes using Illumina NextSeq. We share the draft genome assemblies for strains *Bacillus wiedmanii_*SC129, *Bacillus pseudomycoides*_SC131, *Bacillus pumilis*_SC133, *Peribacillus butanolivorans_*SC135, *Bacillus thuringiensis_*SC136, *Priestia megaterium_*SC138, and *Bacillus wiedmanii_*SC141. Draft genome sizes range from 3,645,032 to 5,969,865 bp, with GC content between 34.8% and 41.2%.

## ANNOUNCEMENT

Endospore-forming bacteria are frequently found in a variety of environments, including terrestrial soils, and many are considered to be plant growth-promoting (1). We collected two soil cores on January 23, 2024 from wooded areas of a suburban college campus and separated mineral and organic horizons from each core (Table 1 [2]). Strain *Bacillus wiedmannii* SC129 was isolated from the organic horizon, and strains *Priestia megaterium* SC138 and *Bacillus wiedmannii* SC141 were from the mineral horizon from location 42.7205,–73.7534, which is on a wooded slope between campus buildings. Strains *Bacillus pumilus* SC133 and *Peribacillus butanolivorans* SC135 were isolated from the organic soil horizon, and strains *Bacillus pseudomycoides* SC131 and *Bacillus thuringiensis* SC136 were isolated from the mineral horizon from location 42.7199,–73.7488, which is a wooded area near a small wetland. We isolated the individual endospore-forming bacterial strains by mixing approximately 100 µL of soil with 1 mL of sterile water, vortexing the samples, heating them to 95°C for 10 minutes to ensure the presence of endospores, and then spreading the suspended cells onto tryptic soy agar + 5% sheep blood using a sterile swab. We incubated the plates overnight at 37°C as described previously (3, 4). Isolates were colony purified, and tryptic soy broth cultures were grown for approximately 4 hours. Then DNA was isolated using a Promega DNA Wizard Kit following the digestion of peptidoglycan with 1.15 µg/µL lysozyme in 50 mM EDTA.

**TABLE 1:** Summary of genome data and accession information

Strain	SC129	SC131	SC133	SC135	SC136	SC138	SC141
Site	42.7204552,–73.7533970	42.7198559,–73.7488097	42.7198559,–73.7488097	42.7198559,–73.7488097	42.7198559,–73.7488097	42.7204552,–73.7533970	42.7204552,–73.7533970
Soil horizon	Organic	Mineral	Organic	Organic	Mineral	Mineral	Mineral
Species	*Bacillus wiedmannii*	*Bacillus pseudomycoides*	*Bacillus pumilus*	*Peribacillus butanolivorans*	*Bacillus thuringiensis*	*Priestia megaterium*	*Bacillus wiedmannii*
Number of reads	5,545,784	6,877,022	6,439,688	6,974,014	6,648,606	5,923,568	5,481,716
Number of contigs	31	100	40	46	31	41	27
Total length (bp)	5,485,886	5,216,294	3,645,032	5,859,099	5,969,865	5,853,104	5,671,044
*N*_50_	781,638	195,779	218,892	299,378	634,513	903,089	927,123
% GC	35.2	35.5	41.2	37.9	34.8	37.5	35.1
Predicted genes (Prokka via KBase)	5,742	5,258	3,695	5,635	5,953	6,063	5,762
Predicted genes (GenBank)	5,639	5,387	3,706	5,771	6,062	6,084	5,861
Number of predicted secondary operons (antiSMASH)	12	12	10	8	16	8	10
Predicted natural product types (specific known cluster name and similarity)	Terpene (molybdenum cofactor 17%), 4 NRPS (anachelin 10% and bacillibactin 85%), 3 RiPP-like, betalactone (fengycin 40%), LAP, NI-siderophore (petrobactin 100%), cyclic lactone autoinducer/thiopeptide	Lassopeptide (paeninodin 100%), LAP, 5 NRPS (bacillibactin 85% and desmamide A/B/C 18%), lanthipeptide class II (plantaricin W 66%), terpene, betalactone (fengycin 40%), 2 RiPP-like	Two betalactone (fengycin 53%), other (bacilysin 85%), T1PKS (zwittermicin A 18%), NRPS (lichenysin 92%), NI-siderophore (schizokinen 60%), terpene, T3PKS, RRE containing, RiPP-like	Two terpenes, two lassopeptide (paeninodin 100%), betalactone (fengycin 46%), NI-siderophore (schizokinen 60%), LAP, T3PKS	LAP, 6 NRPS (bacillibactin 85%), NI-siderophore (petrobactin 100%), betalactone (fengycin 40%), 2 RiPP-like, NRPS-like, terpene (molybdenum cofactor 17%), ranthipeptide, 2 RRE containing (thuricin CD 100%)	Three terpenes (50% carotenoid and 13% surfactin), phosphonate, RRE-containing, T3PKS, NI-siderophore (62% schizokinen), lanthipeptide class I	LAP, 3 NRPS (bacillibactin 85%), NI-siderophore (petrobactin, 100%), betalactone (fengycin 40%), lassopeptide (paeninodin 100%), terpene, 2 RiPP-like
KBase narrative	https://kbase.us/n/172257/32/	https://kbase.us/n/172258/23/	https://kbase.us/n/172259/45/	https://kbase.us/n/172260/62/	https://kbase.us/n/172261/23/	https://kbase.us/n/172262/51/	https://kbase.us/n/172263/26/
BioProject accession	PRJNA862062	PRJNA862062	PRJNA862062	PRJNA862062	PRJNA862062	PRJNA862062	PRJNA862062
SRA accession	SRX25775142	SRX25775143	SRX25775144	SRX25775145	SRX25775146	SRX25775147	SRX25775148
GenBank accession	JBLOJC000000000	JBLOJB000000000	JBLOJA000000000	JBLOIZ000000000	JBLOIY000000000	JBLOIX000000000	JBLOIW000000000

Genomic DNA libraries were prepared using the tagmentation-based Illumina DNA Prep Kit and IDT 10 bp unique dual indices and sequenced as paired-end 151 bp reads by SeqCenter (Pittsburgh, PA, USA) using an Illumina NovaSeq X Plus sequencer, who processed reads with bcl-convert version 4.2.4. Sequence reads from each isolate were imported into separate narratives in the KBase environment for analysis (5–7). We checked the read quality with FastQC version 0.12.1, trimmed reads with Trimmomatic version 0.36, assembled genomes *de novo* using SPAdes version 3.15.3, and annotated the assemblies using RASTtk version 1.073, Prokka version 1.14.5, and DRAM version 0.1.2, and PGAP via GenBank. We determined probable species identities using TYGS and GTDB-Tk, and the results were concordant between programs (Table 1 [8–12]). All programs were run using default parameters. We also ran the draft genome assemblies through the antiSMASH version 7.0 secondary metabolite prediction program (13, 14).

Draft genomes range in size from 3,645,032 to 5,969,865 bp and from 34.8% to 41.2% GC. Each strain is predicted to make 8–16 unique secondary metabolites, including terpenes, RiPPs, non-ribosomally synthesized peptides, and NI-siderophores, among others (Table 1). While many strains are predicted to make at least one well-characterized natural product, including paeninodin and petrobactin, many operons identified have low or no similarity to known secondary metabolites (Table 1 [15–17]). Therefore, these strains and genomes represent useful additions to our knowledge of soil microbes and potential sources of beneficial natural products.

## Data Availability

Sequence data are available via KBase (umbrella narrative https://kbase.us/n/189485/18/ DOI: 10.25982/189485.18/2538685). Raw reads are available in the SRA and genome drafts in GenBank under BioProject PRJNA862062 (Table 1).
